# Examination of Weight-Loss Motivators and Family Factors in Relation to Weight Management Strategies and Dietary Behaviors among Adolescents with Obesity

**DOI:** 10.3390/nu13051729

**Published:** 2021-05-20

**Authors:** Bridget K. Biggs, Dawn K. Wilson, Mary Quattlebaum, Seema Kumar, Alicia Meek, Teresa B. Jensen

**Affiliations:** 1 Department of Psychiatry & Psychology, Mayo Clinic, Rochester, MN 55905, USA; 2 Department of Psychology, University of South Carolina, Columbia, SC 29208, USA; wilsondk@mailbox.sc.edu (D.K.W.); MJQ@email.sc.edu (M.Q.); 3 Division of Pediatric Endocrinology and Metabolism, Department of Pediatric and Adolescent Medicine, Mayo Clinic, Rochester, MN 55905, USA; Kumar.Seema@mayo.edu; 4 Department of Employee and Community Health, Mayo Clinic, Rochester, MN 55905, USA; Meek.Alicia@mayo.edu; 5 Department of Family Medicine, Mayo Clinic, Rochester, MN 55905, USA; Jensen.Teresa@mayo.edu

**Keywords:** motivation, dietary habits, weight management, obesity, adolescence, family factors

## Abstract

The study aim was to test hypotheses informed by self-determination theory (SDT) regarding associations of adolescent motivators for weight loss and family feeding practices on understanding adolescent weight management and dietary behaviors. Adolescents (*n* = 71) with obesity were recruited from a large medical center in the Midwest USA and completed questionnaire assessments via an online survey. Results supported hypotheses that endorsement of health motivators for weight loss, conceptualized as autonomous (intrinsic) motivation, and positive family support would be associated with healthier weight management practices and dietary behaviors. Nuanced findings related to social- and self-esteem-related motivators for weight loss indicated a need for further understanding of these weight-loss motivators in the context of SDT. The current study findings highlight the importance of addressing motivational factors and family influences in research and practice related to promoting healthy dietary habits and weight management strategies among adolescents with obesity.

## 1. Introduction

Adolescent obesity in the U.S. has escalated, with recent prevalence estimate of 20.6% [1]. Nearly half of youth in the U.S. are attempting to lose weight [2,3]. While some adolescents attempt to lose weight by engaging in healthy weight control practices (e.g., healthy diet, physical activity), others control their weight through unhealthy practices that increase risk for further weight gain and disordered eating (e.g., skipping meals, fasting, eating little, taking diet pills) [4,5]. Poor dietary intake, including sweetened beverage and fast-food intake, is a leading risk factor for adolescent obesity [6,7], and dietary modifications such as reducing sugar and fat intake and increasing fruit and vegetable consumption are common intervention targets. Adolescents’ weight control practices and dietary intake may be shaped by their personal reasons for pursuing weight loss as well as by family feeding practices; however few studies have conjointly examined motivators and family factors as they relate to adolescents’ weight-related health behaviors.

Prior research has demonstrated that adolescents’ most common reasons for pursuing weight loss include health, social, appearance, and self-esteem motives [8]. Although the literature on adolescent weight loss motivators has been primarily descriptive and atheoretical, Self Determination Theory (SDT) [9] could be a useful framework for organizing prior research findings and generating hypotheses. Specifically, SDT provides a framework for conceptualizing the influence of motivation and social factors on the initiation and maintenance of health behaviors. According to SDT, autonomous motivation is characterized by intrinsic drive, novelty, and enjoyment, whereas controlled motivation is defined as driven by external forces or social pressures in obtaining a certain outcome [9,10]. Autonomous motivation has been linked to positive health outcomes in youth, including improved diet quality, physical activity, weight loss, as well as sustained healthy behavior change [11,12,13,14]. In contrast, controlled motivation has been associated with poor dietary intake and has been associated with only short-term changes in dietary behaviors [15,16]. These distinct types of motivation are important to consider in relation to adolescents’ reasons for weight loss. Motivations for weight loss rooted in a desire to improve health (e.g., better health, avoid health problems) may be characterized as intrinsic or autonomous motivation, while social factors (e.g., reduce teasing, making friends) and appearance (e.g., looking better) may be characterized as extrinsic or controlled motivation.

Results from prior studies are consistent with this conceptualization. For example, a study including children and adolescents found that health-related motivators for weight loss (e.g., desire to improve sports performance) were associated with healthy weight control behaviors, including efforts to exercise and decrease sweets and fat intake [2]. In contrast, these investigators also found that social motivators (e.g., teasing, peers trying to lose weight) and appearance (e.g., desire to look better) were related to unhealthy weight management strategies, including skipping meals and dietary restriction, respectively. Additional research suggests mood and self-esteem-related weight loss motivators are associated with preoccupation with being over-weight and more dieting [17]. Taken together, these studies suggest that health-related motivators may operate as autonomous motivators that promote healthier weight management strategies and dietary practices and that social, appearance, and self-esteem reasons for weight loss may operate as controlled motivation leading to less desirable outcomes. In contrast, a few qualitative studies suggest that self-esteem and social motivators may be linked to positive health outcomes [18,19]. Specifically, a desire to improve self-esteem has been identified as a facilitator or motivator, not a barrier, for healthy eating among young adults [18]. Further, social motivators for healthy eating and physical activity have been associated with healthier outcomes in young adults especially under supportive conditions (e.g., social inclusion, positive family climate) [19]. Further research is needed to better understand the relationship between weight loss motivators and associated health behavior outcomes especially among adolescents with obesity, who are at increased risk for developing chronic health conditions and eating disorders [5,20].

In addition to adolescents’ personal motivators for weight loss, familial and parental factors have important implications on adolescents’ health behaviors. Based in Family Systems Theory (FST), prior research has shown supportive and nurturing family interactions and parenting practices promote healthy behavioral outcomes, including healthier weight management practices and improved dietary habits among adolescents [5,21,22,23,24]. Further, parental support for healthy eating has been associated with healthier dietary intake (e.g., increased fruit and vegetable intake, reduced sweetened beverages), as well as sustained weight loss among adolescents [21,25] In contrast, more restrictive family feeding practices (restricting child’s access to foods), are associated with poorer dietary outcomes [26,27,28].

The purpose of the current study was to examine whether adolescents’ endorsement of various motivators, as well as family practices related to adolescents’ eating behaviors, were predictive of weight management practices and dietary habits of adolescents with obesity. It is important to examine adolescents’ approach to weight management separate from dietary habits, as weight management strategies are relevant to adolescents’ risk for disordered eating and longitudinal weight trajectories and dietary habits are associated with weight gain/loss. Further, approach (e.g., reducing sugar intake) may differ from habits (e.g., high sugar intake vs. already reduced sugar intake). The study examined the following hypotheses: (1) endorsement of health motivators—conceptualized as autonomous motivation—would be associated with more healthy weight management strategies, less unhealthy weight management strategies, and healthier eating habits (i.e., less fast food, unhealthy snacks, and sugary foods, more fruits and vegetables), (2) endorsement of appearance, self-esteem, and social motivators—conceptualized as controlled motivation—would be associated with less healthy weight management practices, more unhealthy weight management practices, and less healthy eating habits, (3) supportive (versus restrictive) parenting practices would be associated with healthier weight management practices, less unhealthy weight management practices, and healthier dietary habits.

## 2. Materials and Methods

### 2.1. Participants

The study participants were 71 adolescents with obesity and their parents drawn from a larger study investigating motivation and social support among adolescents with obesity. The recruitment strategy aimed to include adolescents with obesity who may or may not be treatment seeking. All participants were recruited from a large medical practice in the Midwest, USA, following multiple recruitment strategies including mailed invitation letters to adolescents identified through an electronic medical record search as meeting inclusion criteria (all 1310 identified were mailed invitations), advertisement in the medical center’s employee newsletter, and referral from the medical center’s pediatric weight management clinic and primary care providers. Adolescent inclusion criteria were: ages 13–17, body mass index (BMI) ≥ 95th percentile for sex and age [29], and ability to complete questionnaires in English, exclusion criteria included a diagnosed active eating disorder in the adolescent’s electronic medical record. Parent inclusion criteria were: parent or guardian of participating adolescent, self-identification as overseeing the family’s eating and physical activity habits, and ability to complete the questionnaire in English. A total of 116 adolescents assented with parental consent and 15 were withdrawn (5 refused further participation, 8 lost contact, 6 were ineligible due to not meeting full study criteria). One-hundred adolescents completed the survey, of which 97 had a parent complete the parent survey. The final sample for the current study consisted of the 71 adolescent participants whose parents participated and who indicated in the survey they were currently trying to lose weight, as questions about motivation for weight loss were presented only to these participants.

### 2.2. Procedures

The Mayo Clinic’s Institutional Review Board approved all study procedures and materials (IRB# 16-008367). After discussing the study with the research coordinator, participants provided written informed consent/assent by mail for all study procedures including completion of a survey and recording information such as height and weight from the adolescents’ medical record. Participants subsequently completed questionnaires via a secure Internet link and received $20 remuneration. Participants were offered the option of completing a paper version of the study questionnaire battery. Additional data were obtained from electronic medical review.

### 2.3. Measures

#### 2.3.1. Demographic Questions

Adolescents provided information about their gender identification, natal-assigned sex, age, and racial and ethnic identification. Parents provided information regarding their gender, racial, and ethnic identification; relationship with the participating adolescent (i.e., mother, father, other); relationship status with the adolescent’s other parent; and highest parental educational attainment as a proxy for socio-economic status. They also answered questions regarding who takes responsibility for groceries and food preparation in the home and for supervising the adolescent’s extracurricular activities.

#### 2.3.2. Family Healthy Eating Support

The Family Healthy Eating (HE) Support scale from the Support for Healthy Lifestyle Questionnaire for Adolescents (SHeL) [30] assessed social support from family members for healthy eating habits. The scale’s seven items consist of statements describing supportive behavior (e.g., “Complimented or praised you on your efforts to eat healthier”) to which participants indicate the frequency to which family members have demonstrated the behavior in the last month on a scale from never (0) to almost always (4). Prior research has supported the content validity (i.e., item generation through focus groups), factor structure, and internal consistency of the SHeL and has demonstrated that the Family HE Support scale is associated with family support for fruit and vegetable intake as well as adolescent-reported fruit and vegetable intake and eating habits [30]. The average of the seven responses served as the Family HE Support score. Internal consistency in this sample was α = 0.88.

#### 2.3.3. Parental Food Controlling Practices

Parents responded to items drawn from the Child Feeding Questionnaire (CFQ) [31], an instrument used in prior research on food-related parenting practices with adolescents to assess their beliefs and behaviors related to the need to restrict their adolescents’ eating. The six items consist of statements (e.g., “I have to be sure that my child does not eat too many high fat foods”), to which participants indicate their degree of agreement from disagree (0) to agree (4). The average of the six ratings served as the Parental Dietary Restriction score. Prior research has supported the CFQ’s factor structure, internal consistency, and association with child BMI in an adolescent sample [31]. Internal consistency in this sample was α = 0.88.

#### 2.3.4. Motivators for Adolescent Weight Loss

Adolescents’ motivators for weight loss were assessed with the Motivators for Adolescent Weight Loss Questionnaire [32]. If adolescent participants indicated on the study survey that they were trying to lose weight, they were presented with the prompt: “How important TO YOU are each of the following reasons for you to lose weight?” followed by a list of 10 reasons to rate on a scale from not at all important (0) to extremely important (10). Reasons included “to have better health”, “to avoid health problems”, “to look better”, “to feel better about myself”, “to be ready for an upcoming event (prom/dance, graduation, wedding, vacation, etc.)”, “to improve my social life (e.g., reduce teasing, bullying, be better accepted, make friends, etc.)”, “to do better at school”, “to do better at sports”, “to have better energy”, and “to feel better physically (e.g., less pain).” Due to high correlation between the two health items, “to have better health” and “to avoid health problems” (r = 0.73), these items were combined in a single health motivation score. In light of the current study’s theoretical lens, analyses focused on the Health Motivation score as autonomous motivation and the Appearance (“to look better”), Self-Esteem (“to feel better about myself”), and Social (“to improve my social life”) Motivation scores as controlled motivation. Prior research with this instrument has indicated good content coverage of adolescents’ reasons for trying to lose weight [32].

#### 2.3.5. Adolescent Healthy and Unhealthy Weight Management Strategies

Adolescents reported the frequency they engaged in six healthy weight management behaviors (i.e., exercise, ate more fruits and vegetables, ate less high-fat foods, ate less sweets, drank less soda pop, watched portion sizes) and 10 unhealthy weight management behaviors (i.e., fasted, ate very little food, took diet pills, vomited intentionally, used laxatives, used diuretics, used food substitutes, skipped meals, smoked cigarettes, followed a special diet) [4]. Response options were modified from the original yes/no format to indicate frequency: ranging from never (0), to on a regular basis (3). Scale scores for healthy weight management and unhealthy weight management were means across the six- and ten-item responses, respectively. Internal consistency for the scales in this sample were α = 0.78 for healthy weight management and α = 0.76 for unhealthy weight management. Supporting their predictive validity, prior research using these scales have found endorsement of healthy weight management strategies predictive of successful weight loss among youth with overweight [33] and unhealthy weight management strategies predictive of later overweight status, binge eating, and extreme weight control practices [5].

#### 2.3.6. Adolescent Dietary Behaviors

Adolescents reported consumption of sweetened beverages, sweet and salty snacks, and fruits and vegetables in the last week in response to items from the Beverage and Snack Questionnaire 2 [34]. For each item, respondents indicate the frequency they consumed the type of food presented in the past week using a 7-point Likert scale, which was scored according to guidelines from the instrument’s authors, i.e., ever/less than 1 per week (0), 1 per week (1), 2–4 per week (3), 5–6 per week (5.5), 1 per day (7), 2–3 per day (17.5), and 4+ times/week (24). For this study and consistent with author scoring guidelines, scores for Sweetened Beverage Consumption were the five-item sum for fruit-flavored drinks, sports drinks, regular soda/pop, energy drinks, and sweetened coffee/tea drinks. Scores for Sweet and Salty Snack Consumption were the six-item sum for chips, other salty snacks, candy, pastries, baked sweets, and ice cream. Scores for Fruit and Vegetable Consumption were the two-item sum for fruits and vegetables. The BSQ has demonstrated good criterion validity, with correspondence of individual food items reported on the BSQ and 4-day dietary recall ranging from r = 0.56 to 0.87, and 4–6-week test-retest reliability ranging from r = 0.72 for sweets/snacks to 0.85 for fruits and vegetables Adolescents also reported their fast food consumption in the last week in response to single item, “In the past week, how often did you eat something from a fast-food restaurant (like McDonald’s, Burger King, Hardee’s, etc.)?” using a 5-point Likert scale scored according to survey author’s guidelines: never (0), 1–2 days (1.5), 3–4 days (3.5), 5–6 days (5.5), everyday (7). Construct validity has been supported by correspondence of young adults’ responses to this item to their reports of total caloric intake and consumption of fats, sodium, French fries, and soft drinks [35].

### 2.4. Data Analytic Plan

Statistical analyses were completed with IBM SPSS Statistics 25. Descriptive statistics were calculated to describe the demographic characteristics of the sample. Bivariate correlations assessed associations among demographic, family influence, and motivational factors with dietary behaviors and weight management practices. Hierarchical linear regressions tested hypotheses regarding unique associations of the two, family influence scales (Family HE Support, Parental Dietary Restriction) and four motivation ratings (Health, Appearance, Self-Esteem, Social) with adolescents’ weight management practices (Healthy and Unhealthy) and dietary consumption (Sweetened Beverages, Fast Food, Sweet and Salty Snacks, Fruit and Vegetables). Specifically, six hierarchical regressions were conducted, one for each of these outcome variables. In Step 1, BMI z-score (zBMI), age, gender, and parental education were entered as covariates, given known associations of these variables with dietary habits. The family influence variables were standardized into z-scores and entered in Step 2, and motivation ratings were standardized into z-scores and entered in Step 3. The F-statistic and t-tests were used to test statistical significance of each model step and unique contributions of each independent variable, respectively. To reduce type 1 error, independent variable statistics were interpreted only when the F change test for the model step taken together was significant. Data were complete except for one participant who was missing BMI data within the specified time frame around the study participation date. This missing data point was handled with mean substitution within the regression model specification, consistent with recommendations to use substitution methods for minor (i.e., <2%) item-level nonresponse [36]. Across regression models, variance inflation factors (VIF) and tolerance statistics were examined for evidence of multicollinearity, with all statistics within acceptable ranges. Scatterplots of standardized residuals were examined to confirm assumptions of normality and homoscedasticity were met. Potentially influential scores were identified as standardized residuals greater than 3.0. Four scores met this criterion, all from different participants, and all falling at the high end of the distribution. Raw scores were 53.0 for sugary beverages, 33.5 for sugary and salty snacks, 48 for fruit and vegetables, and 2.0 for unhealthy weight control. Because these scores are plausible though rare, they were retained unaltered following best practices recommendations for multiple regression analyses [37].

## 3. Results

### 3.1. Description of Participants

Table 1 summarizes demographic information as well as means and standard deviations for the primary study variables among the study sample. The sample mean for zBMI was more than 2 standard deviations above average by age and gender based on national standards [29]. Age was evenly distributed, and there were approximately equal numbers of males and females. Adolescents and their parents predominantly identified as White/Caucasian. Most parents had attained a bachelor’s degree or higher in education and were living in the same household with the other parent. Most parents took primary responsibility (*n* = 69, 71%) or shared responsibility (*n* = 26, 27%) for household food purchasing and preparation. Most took primary responsibility (*n* = 42, 43%) or shared responsibility (*n* = 28, 29%) for supervising the adolescents’ extracurricular activities. About a quarter (*n* = 26, 27%) of parents reported adolescents engaged in activities on their own.

### 3.2. Bivariate Correlations

Table 2 summarizes bivariate correlations of demographic, family, and motivation variables with adolescent dietary behavior and use of weight management strategies. Health motivation was associated with less intake of sweetened beverages, fast food, and salty and sugary snacks and with less frequent use of unhealthy weight management strategies. Although motivation to improve one’s social life was positively correlated with fast food and salty, sugary snack intake, and social motivation was also positively associated with healthy weight management strategies. Self-esteem was also related to healthy dietary behavior, specifically fewer fast-food visits. In terms of family factors, the only significant bivariate correlation was consistent with autonomous motivation predictions; namely, family support of healthy eating was positively associated with adolescents’ engagement in healthy weight management strategies.

### 3.3. Predicting Healthy Weight Management Strategies

As summarized in Table 3, the first step of the hierarchical regression model regressing use of healthy weight management strategies on demographic co-variates yielded insignificant F change. Step 2 examining contribution of family factors was significant according to the F-change statistic. Within that step, family healthy eating support was significantly and positively associated with adolescents’ use of healthy strategies for weight management. Step 3 examining motivators for weight loss was marginally significant (*p* = 0.06). Within that step, social motivation was significantly and positively associated with use of healthy weight management strategies, and appearance motivation was inversely related to use of healthy weight management strategies at a marginally significant level (*p* = 0.06).

### 3.4. Predicting Unhealthy Weight Management Strategies

No steps of the hierarchical regression analyses related to use of unhealthy weight management strategies were statistically significant. Although the omnibus test for Model 3 did not reach significance, health motivation specifically was significantly and inversely related to unhealthy weight management strategies (*p* < 0.05). Detailed results are presented in Table S1

### 3.5. Predicting Fast Food Consumption

As summarized in Table 4, the first and second steps of the hierarchical regression models regressing fast food consumption on demographic covariates and family factors yielded insignificant F change. Step 3 evaluating weight loss motivators was significant. Motivation to lose weight for health and self-esteem reasons were uniquely and inversely related to fast food consumption at a marginally significant level (*p* = 0.07 and *p* = 0.08, respectively).

### 3.6. PredictingSweetened Beverage Consumption

As shown in Table 5, the first step of the hierarchical regression analyses indicated demographic variables accounted for a significant amount of variance (17%) in sweetened beverage consumption. Higher levels of sweetened beverage consumption was associated with male gender and lower parental education. The second step was marginally significant (*p* = 0.07). Parental dietary restriction was positively associated with sweetened beverage consumption (*p* = 0.05). The third step examining adolescent weight loss motivators yielded an insignificant F change.

### 3.7. Predicting Salty and Sugary Snack Consumption

For salty and sugary snacks, steps 1 and 2 yielded insignificant F-change (ΔF = 0.60, *p* = 0.67, ΔR^2^ = 0.04 for Step 1; ΔF = 1.65, *p* = 0.20, ΔR^2^ = 0.05 for Step 2). Step 3 examining motivators was marginally significant (ΔF = 2.15, *p* = 0.09, ΔR^2^ = 0.12). Health motivation was significant and inversely related salty and sugary snack consumption (β = −0.27; *p* = 0.05). Detailed results are presented in Table S2.

### 3.8. Predicting Fruit and Vegetable Consumption

No steps of the hierarchical regression analyses related to fruit and vegetable consumption were statistically significant. Detailed results are presented in Table S3.

## 4. Discussion

The aim of this study was to examine the associations of adolescent motivators for weight loss and family feeding practices on adolescent weight management and dietary behaviors. Results were largely consistent with the SDT framework. Specifically, health motivation for weight loss, conceptualized as autonomous motivation, was associated with healthier eating habits (i.e., less fast food and salty/sugary snack consumption) and weight management practices, and family healthy eating support was related to adolescents’ healthy weight management practices. Nuanced findings related to social- and self-esteem-related motivations for weight loss indicated a need for further understanding of these weight-loss motivators in the context of SDT. First, social motivation was associated with greater consumption of salty/sugary snacks and fast food, consistent with SDT-informed hypotheses, but was also positively related to healthy weight management practices. Second, contrary to SDT predictions, self-esteem-related motivation was related to less frequent fast-food visits. This study is among the first to examine adolescents’ reasons for desiring weight loss and family factors in the same model as a robust test of their unique relations with dietary and weight management behaviors of adolescents with obesity. The study expands upon prior research that has examined weight loss motivators and family factors separately and not in severely obese adolescents, and further elevates this line of research by testing hypotheses informed by SDT.

An important set of findings from this study was that adolescents with health-related motivation for weight loss approached weight management in a less unhealthy way and reported healthier eating habits. Specifically, adolescents who more strongly endorsed health reasons for pursuing weight loss reported less consumption of fast food, sugary and salty snacks (often referred to as “junk food”), and sugar-sweetened beverages. The findings related to unhealthy weight management, fast food, and snacks were particularly robust, as they emerged in both bivariate and multivariate analyses. Prior research has found that, among adults enrolled in a behavioral weight loss intervention, concern for one’s health was predictive of program completion and meeting weight loss goals [38], suggesting greater commitment to health behavior change when health reasons are driving intrinsic motivation. These findings are consistent with our conceptualization of health motivators as autonomous motivation (intrinsic) as well as prior research demonstrating connections between autonomous motivation and successful engagement of adults in weight loss intervention [39]. These findings are important because they highlight the relevance of health-related motivation as a possible protective factor against disordered weight management strategies as well as engagement in healthier dietary choices. Viewing these findings through the lens of SDT-informed health promotion research further emphasizes the importance of autonomous motivation and autonomy support [40], that is, that adolescents themselves see weight loss as a means to better health and value health as an outcome as opposed to their actions being driven by social norms or others’ expectations.

In terms of social context, the present study found that adolescents were more likely to engage in healthy weight management practices (e.g., attending to portion sizes and nutritional content of foods, engaging in exercise) when they described their family as engaging in supportive behaviors for healthy eating (e.g., encouraging healthy eating choices, preparing healthy meals and snacks, modeling healthy eating habits). Previous studies have established that family social support of healthy eating is associated with healthy weight management practices, including healthier eating habits, among adolescents [25,30], and that adolescents see parents as the primary source of healthy eating support within the family [41]. Importantly, the current study demonstrated this association to be robust after controlling for demographic variables linked to pediatric obesity. Engagement in unhealthy and extreme weight management practices, such as dietary restriction and skipping meals, are known to predict excessive weight gain over time and are more likely to be used by adolescents who are overweight or obese [42]. On the other hand, healthy weight management practices consistent with lifestyle interventions for obesity are protective against excess weight gain, as demonstrated in longitudinal research following participants from adolescence to adulthood [42]. The current study findings provide evidence supporting the association between parents’ engagement in autonomy supportive behaviors and adolescent healthy diet as part of adolescent obesity prevention and intervention programs.

Current study findings related to social motivation were more nuanced. Adolescents motivated to lose weight for social reasons consumed more salty/sugary snacks and fast food, consistent with SDT-informed hypotheses that control motivation may interfere with enacting healthy behaviors. Social motivation was also positively related to healthy weight management practices. These mixed findings fit with the SDT concept of introjected regulation, i.e., motivation driven by externally held values that individuals have partially integrated into their value system [40]. Research suggests that introjected regulation may lead to short term engagement in healthy action but carry longer term negative effects on emotional well-being [40]. It is possible that adolescents who are motivated by social goals may engage in healthy behaviors with the intention of losing weight to improve their social life and struggle to sustain their efforts if either or both desired outcomes are not realized. Another possible interpretation of the current study findings is that adolescents who are socially-driven to lose weight are autonomously motivated, increasing the likelihood of a number of healthy behaviors, but that adolescents who strongly value social connection are also more likely to find themselves in social settings with tempting food choices. Prior research has shown correlations between adolescent friends in their frequency of fast-food visits, with higher associations occurring in later adolescence [43] and qualitative work points to ubiquitous availability of calorically dense food in social settings as a barrier to healthy eating among young adults [19]. The observation that social motivation was significantly associated with fast food and sugary/sweet snack intake at the bivariate level but not in multivariate analyses suggests that other motivators for weight loss may be more relevant to “junk” food consumption. Namely, health and self-esteem motivation emerged as consistently related to fast food visits, and health motivation emerged as consistently associated with sugary/salty snack consumption. The valence of social context as a potential influence on the association between social motivation and health behaviors could be a fruitful direction for future research, as some social motivators for health behaviors may be based in a more supportive context (e.g., supportive family environment, peer inclusion), whereas others may be rooted in a more controlling context (e.g., societal pressure, unhealthy family peer influence) [19].

In the current study, the desire to lose weight “to feel better about myself” was associated with less frequent fast-food visits, suggesting self-esteem driven motivation may have acted as a facilitator of healthy eating in this study rather than as a barrier, as would be predicted when self-esteem is conceptualized as a controlled motivator. Taken at face value, motivation “to feel better about myself” could be interpreted as coming from a need to improve self-esteem based on currently low self-worth or as coming from an internalization of the thin ideal, with both of these possibilities connected with controlled motivation in the SDT framework [9]. In fact, low self-esteem has been found to mediate the relation between low autonomy and disordered eating behaviors in young women [44], and motivation to improve self-esteem does tend to cluster with controlled motivators such as a desire to look better [45]. However, the findings from this study suggest that a desire to lose weight in order to feel better about oneself could function as an intrinsic goal, or at least be associated with introjection (aligning with one’s self-concept), with the function of this goal perhaps depending on the degree to which basic needs of autonomy, self-competency, and relatedness have been met [18,46]. Consistent with this interpretation, one experimental study found that when individuals were taught to engage in self-compassion, higher induced body dissatisfaction was associated with higher motivation for self-improvement [47]. It could also be that adolescents’ endorsement of self-esteem motivation for weight loss reflected their valuing self-worth and actions to promote it, thus facilitating autonomous motivation.

Hypotheses related to appearance motivation were not supported. Appearance motivation was not related to weight management strategies nor dietary behaviors at the bivariate level, suggesting that the trend-level relationship with healthy weight management strategies in the multivariate regression model was likely spurious. This study’s results stand in contrast to prior studies that have linked appearance motivation for weight loss with poorer health and weight outcomes among adults [38,48] and, more broadly, have connected controlled motivation with poorer outcomes related to health behaviors [45]. Understanding connections between appearance-related motivation for weight loss and health behaviors is particularly relevant in adolescence, given the elevated importance of body image during this developmental period as well as the increased likelihood of body dissatisfaction among adolescents who experience weight-related stigma and bullying [49]. Prior research has linked appearance-related weight-loss motivation with extreme dieting behaviors among adults, and body dissatisfaction is one of the strongest predictors of extreme weight management practices among adolescents [49,50]. It is possible that the exclusion of adolescents with an eating disorder may have truncated variability in reported unhealthy weight control behaviors in this study.

### Limitations and Future Directions

As with any correlational study, causation cannot be inferred from the current study. The study aimed to reduce potential for erroneously interpreting spurious findings by specifying theory-informed hypotheses and including important weight-related demographic variables in the statistical models. Future research with longitudinal designs would provide a stronger test of direction of effects. The study’s modest sample size may have limited detection of small effects; at the same time, statistically significant findings can be considered robust given the study’s power and conservative approach to hypothesis testing (i.e., inclusion of control variables and omnibus F-change statistic in hierarchical regression). The participation rate relative to the number of study invitations sent is low and potentially selective. Specifically, study participants identified predominantly White/Caucasian and many parents reported post-secondary education, reflective of county population statistics for the study location [51]. At the same time, this recruitment strategy successfully captured a sample that represented variability in obesity treatment status. Replication of study findings across diverse samples would inform the degree to which findings can be generalized across populations. Self-report assessments of health behaviors, including food frequency questionnaires, are subject to error [52]. Our team opted for self-report methods as a low-burden, low-cost option for initial hypothesis testing. Replication of this study with “gold standard” multi-pass 24-h dietary recall methods would contribute additional confidence in current findings.

## 5. Conclusions

The study is among the first to examine adolescents’ motivations for weight loss and family factors in the same model as a robust test of their unique relations with dietary and weight management behaviors of adolescents with obesity. Findings supported hypotheses derived from SDT that health-focused motivation—conceptualized as autonomous motivation—and positive family support would be associated with healthier dietary and weight management behaviors among adolescents with obesity. Nuanced findings related to social and self-esteem motivators as well as non-support for hypotheses related to appearance motivation indicated a need for further understanding of these weight-loss motivators in the context of SDT. Together, current study findings highlight the importance of addressing motivational factors and family influences in research and practice for promoting healthy weight management and dietary behaviors among adolescents with obesity.

## Figures and Tables

**Table 1:** Participant demographics and psychosocial characteristics.

Variable	Mean/Count	Standard Deviation/ Percent of Sample
zBMI	2.38	0.41
Age in Years	15.66	1.60
Gender identity ^1^:		
Female	35	49.3%
Male	36	50.7%
Ethnic/racial identity ^2^		
Hispanic/Latino	2	2.8%
White/Caucasian	65	91.5%
American Indian/Alaskan Native	1	1.4%
Black/African American	4	5.6%
Other Race	1	1.4%
Highest Parent Education		
Some high school	2	2.8%
High school graduate	12	16.9%
Associate’s degree	19	26.8%
Bachelor’s degree	23	32.4%
Graduate/Professional degree	15	21.1%
Family Healthy Eating Support	2.47	0.91
Parental Dietary Restriction	1.90	0.90
Health Motivation	8.43	1.95
Appearance Motivation	8.45	2.08
Self-Esteem Motivation	8.69	1.77
Social Motivation	5.61	3.50
Healthy Weight Management Strategies	2.00	0.58
Unhealthy Weight Management Strategies	0.38	0.37
Fast Food Consumption	1.55	1.41
Sweetened Beverage Consumption	7.25	8.81
Salty and Sugary Snack Consumption	9.06	6.99
Fruit and Vegetable Consumption	13.07	10.96

^1^ Three participants identified as transgender (*n* = 2) or male (*n* = 1) with female natal-assigned sex and were included in analyses as male according to their gender identification. ^2^ Ethnic/racial identity was non-exclusive. zBMI: body mass index z-score.

**Table 2:** Bivariate correlations of demographic, family, and motivation variables with dietary behavior and weight management strategies.

Variable	Healthy WM	Unhealthy WM	Fast Food	Sweetened Beverages	Salty, Sugary Snacks	Fruit and Vegetables
zBMI	0.09	−0.02	0.13	0.21 *	0.07	0.01
Age	−0.03	−0.03	0.06	0.18	−0.04	−0.01
Gender	−0.13	−0.16	0.09	0.27 *	0.17	−0.14
Parent Education	−0.04	−0.02	−0.13	−0.24 *	0.01	0.12
HE Support	0.26 *	−0.12	−0.11	−0.16	−0.12	0.17
Dietary Restriction	−0.14	0.18	0.11	0.18	0.18	−0.10
Health Motivation	0.16	−0.29 **	−0.35 **	−0.24 *	−0.32 **	−0.11
Appearance Motivation	−0.07	0.07	0.03	0.01	0.18	−0.10
Self-Esteem Motivation	0.14	−0.00	−0.21 *	−0.18	−0.08	0.02
Social Motivation	0.20 *	0.09	0.27 *	0.08	0.21 *	−0.14

zBMI: Body Mass Index z-score; HE: Healthy Eating; WM: weight management; * *p* < 0.05, ** *p* < 0.01.

**Table 3:** Regression analyses assessing associations of family factors and weight loss motivators with healthy weight management.

Model/Parameter	B	SE	β	R^2^	ΔR^2^	ΔF Sig
Model 1—Covariates				0.04	0.04	0.65
Intercept	1.73	0.82				
zBMI	0.22	0.19	0.15			
Age	−0.01	0.05	−0.04			
Gender	−0.10	0.07	−0.17			
Parent Education	−0.01	0.07	−0.02			
Model 2 ^a^—Family Influences				0.13	0.09	0.045
Intercept	1.61	0.83				
Healthy Eating Support	0.14	0.07	0.25 *			
Parental Dietary Restriction	−0.11	0.07	−0.18			
Model 3 ^b^—Weight Loss Motivators				0.25	0.12	0.06
Intercept	1.75	0.80				
Health	0.05	0.08	0.09			
Appearance	−0.17	0.09	−0.29 ^†^			
Self-Esteem	0.09	0.09	0.16			
Social	0.22	0.08	0.39 **			

zBMI: Body Mass Index z-score; ^a^ Model 2 included all variables from Model 1. ^b^ Model 3 included all variables from Models 1 and 2; ^†^ *p* < 0.10; * *p* < 0.05, ** *p* < 0.01.

**Table 4:** Regression analyses assessing associations of family factors and weight loss motivators with fast food consumption.

Model/Parameter	B	SE	β	R^2^	ΔR^2^	ΔF Sig
Model 1—Covariates				0.04	0.04	0.62
Intercept	1.29	1.99				
zBMI	0.37	0.46	0.11			
Age	0.00	0.11	0.00			
Gender	0.10	0.18	0.07			
Parent Education	−0.18	0.16	−0.14			
Model 2 ^a^—Family Influences				0.07	0.03	0.41
Intercept	1.30	2.09				
Healthy Eating Support	−0.16	0.17	−0.12			
Parental Dietary Restriction	0.18	0.19	0.12			
Model 3 ^b^—Weight Loss Motivators				0.24	0.18	0.01
Intercept	0.77	1.96				
Health	−0.35	0.19	−0.25 ^†^			
Appearance	0.17	0.21	0.12			
Self-Esteem	−0.39	0.21	−0.27 ^†^			
Social	0.33	0.20	0.23			

zBMI: Body Mass Index z-score; ^a^ Model 2 included all variables from Model 1. ^b^ Model 3 included all variables from Models 1 and 2; ^†^ *p* < 0.10; * *p* < 0.05, ** *p* < 0.01.

**Table 5:** Regression analyses assessing associations of family factors and weight loss motivators with sweetened beverage consumption.

Model/Parameter	B	SE	β	R^2^	ΔR^2^	ΔF Sig
Model 1—Covariates				0.17	0.17	0.01
Intercept	1.98	11.54				
zBMI	2.25	2.64	0.10			
Age	0.46	0.65	0.08			
Gender	2.34	1.04	0.27 *			
Parent Education	−2.10	0.92	−0.26 *			
Model 2 ^a^—Family Influences				0.24	0.07	0.07
Intercept	0.42	11.75				
Healthy Eating Support	−1.24	0.95	−0.15			
Parental Dietary Restriction	2.06	1.04	0.23 ^†^			
Model 3 ^b^—Weight Loss Motivators				0.30	0.06	0.32
Intercept	−2.47	11.78				
Health	−1.34	1.12	−0.15			
Appearance	1.28	1.29	0.15			
Self-Esteem	−1.78	1.28	−0.20			
Social	−0.52	1.19	−0.06			

zBMI: Body Mass Index z-score; ^a^ Model 2 included all variables from Model 1. ^b^ Model 3 included all variables from Models 1 and 2; ^†^ *p* < 0.10; * *p* < 0.05, ** *p* < 0.01.

## Data Availability

The data presented in this study are available on request from the corresponding author. The data are not publicly available according to description of confidentiality and data sharing procedures described in the study’s informed consent and assent documents.

## Supplementary Materials

The following are available online at https://www.mdpi.com/article/10.3390/nu13051729/s1, Table S1: Regression analyses assessing associations of family factors and weight loss motivators with unhealthy weight management strategies; Table S2: Regression analyses assessing associations of family factors and weight loss motivators with salty snack consumption, Table S3: Regression analyses assessing associations of family factors and weight loss motivators with fruit and vegetable consumption..
