# Supplementary material for: Examination of Weight-Loss Motivators and Family Factors in Relation to Weight Management Strategies and Dietary Behaviors among Adolescents with Obesity
Source: Nutrients. 2021 May 20;13(5):1729. doi: 10.3390/nu13051729 (PMC8160946; doi:10.3390/nu13051729)
Supplement: Supplementary file 1 [file nutrients-13-01729-s001.zip › nutrients-1187712-supplementary.pdf]

Table S1. Regression analyses assessing associations of family factors and weight loss motivators with unhealthy weight management strategies.

**Model**/Parameter	B	SE	β	R^2	ΔR^2	ΔF Sig
**Model 1** – Covariates				.03	.03	.77
Intercept	0.41	0.53				
zBMI	0.03	0.12	.04			
Age	-0.01	0.03	-.03			
Gender	-0.06	0.048	-.17			
Parent Education	-0.00	0.04	-.01			
**Model 2** – Family Influences				.08	.05	.20
Intercept	0.38	0.55				
Healthy Eating Support	-0.05	0.05	-.14			
Parental Dietary Restriction	0.07	0.05	.18			
**Model 3** – Weight Loss Motivators				.15	.08	.26
Intercept	0.32	0.55				
Health (autonomous)	-0.12	0.05	-.31*			
Appearance (controlled)	0.03	0.06	.09			
Self-Esteem (controlled)	0.02	0.06	.04			
Social (controlled)	-0.02	0.06	-.06			

zBMI: Body Mass Index z-score; [a] Model 2 included all variables from Model 1. [b] Model 3 included all variables from Models 1 and 2; [†]$p < .10$; *$p < .05$, **$p < .01$.

Table S2. Regression analyses assessing associations of family factors and weight loss motivators with salty snack consumption.

**Model**/Parameter	B	SE	β	R^2	ΔR^2	ΔF Sig
**Model 1** – Covariates				.04	.04	.67
Intercept	13.94	9.88				
zBMI	0.68	2.26	.04			
Age	-0.32	0.56	-.07			
Gender	1.18	0.89	.17			
Parent Education	-.15	0.79	-.02			
**Model 2** – Family Influences				.08	.05	.20
Intercept	12.36	10.25				
Healthy Eating Support	-0.92	0.83	-.14			
Parental Dietary Restriction	1.28	0.91	.18			
**Model 3** – Weight Loss Motivators				.20	.12	.09
Intercept	11.18	9.98				
Health	-1.86	0.94	-.27[†]			
Appearance	1.83	1.10	.26			
Self-Esteem	-1.02	1.09	-.15			
Social	0.28	1.01	.04			

zBMI: Body Mass Index z-score; [a] Model 2 included all variables from Model 1. [b] Model 3 included all variables from Models 1 and 2; [†]$p < .10$; *$p < .05$, **$p < .01$.

Table S3. Regression analyses assessing associations of family factors and weight loss motivators with fruit and vegetable consumption.

**Model**/Parameter	B	SE	β	R^2	ΔR^2	ΔF Sig
**Model 1 –** Covariates				.04	.04	.57
Intercept	4.49	15.43				
zBMI	1.73	3.53	.06			
Age	-.04	0.87	-.01			
Gender	-1.95	1.39	-.18			
Parent Education	1.43	1.24	.14			
**Model 2 –** Family Influences				.09	.05	.22
Intercept	2.53	16.02				
Healthy Eating Support	1.94	1.30	.19			
Parental Dietary Restriction	-1.32	1.42	-.12			
**Model 3 –** Weight Loss Motivators				16	.07	.28
Intercept	0.85	16.01				
Health	-3.06	1.52	-.28*			
Appearance	-0.94	1.76	-.09			
Self-Esteem	1.84	1.74	.17			
Social	-1.80	1.61	-.16			

zBMI: Body Mass Index z-score; [a] Model 2 included all variables from Model 1. [b] Model 3 included all variables from Models 1 and 2; [+]$p < .10$; *$p < .05$, **$p < .01$.
